# The effectiveness of e-mental health interventions on stress, anxiety, and depression among healthcare professionals: a systematic review and meta-analysis

**DOI:** 10.1186/s13643-024-02565-6

**Published:** 2024-05-30

**Authors:** Zemiao Zhang, Yinhuan Hu, Sha Liu, Xiandong Feng, Jinhong Yang, Ling Jie Cheng, Sheena Ramazanu, Xi Vivien Wu

**Affiliations:** 1 School of Medicine and Health Management, Tongji Medical College, Huazhong University of Science and Technology, Wuhan, China; 2 Xiangya School of Nursing, Central South University, Changsha, China; 3 School of Nursing and Midwifery, Queen’s University Belfast, Belfast, UK; 4 Saw Swee Hock School of Public Health, National University of Singapore, Singapore, Singapore; 5 School of Nursing and Health Studies, The Jockey Club Institute of Healthcare (IOH), Hong Kong Metropolitan University, Hong Kong, China; 6 Alice Lee Centre for Nursing Studies, Yong Loo Lin School of Medicine, National University of Singapore, Singapore, Singapore

**Keywords:** Healthcare professionals, Stress, Anxiety, Depression, E-mental health

## Abstract

**Background:**

Many healthcare professionals are experiencing psychological distress. Electronic mental health (e-mental health) interventions are convenient and multifunctional. This review aimed to examine the effectiveness of e-mental health interventions in enhancing the well-being of healthcare professionals and to identify moderating factors.

**Methods:**

A comprehensive and systematic retrieval of randomized controlled trial (RCT) studies was conducted across eight databases. Population, intervention, comparison, and outcome (PICO) were used to define eligibility criteria. Stress, anxiety, and depression were included as the main outcomes. The overall effect was calculated based on the random effect model, and the effect size was presented using the standardized mean difference. The characteristics of the research design, intervention object, and intervention design were further selected as potential moderating factors for subgroup analysis. Meta-regression analyses were finally performed, incorporating intervention duration and sample size as independent variables.

**Results:**

A total of 20 studies were included in the systematic review, and 17 were included in the meta-analysis. A large effect on relieving stress and anxiety and a small-to-medium effect on reducing depression were observed. Subgroup analyses showed that features including mindfulness approaches, online courses, computer use, group interventions, and professional guidance were more favorable in the design of services. Meta-regression revealed that intervention duration only affected anxiety symptoms. Caution should be exercised, as some subgroups had fewer studies and higher heterogeneity. For the secondary outcomes, a large effect on emotional exhaustion and a small-to-medium effect on well-being were observed.

**Conclusion:**

In general, e-mental health interventions significantly improve the psychological health of healthcare staff. Future high-quality, large-scale studies targeting healthcare professionals and specific intervention scenarios are warranted.

**Supplementary Information:**

The online version contains supplementary material available at 10.1186/s13643-024-02565-6.

## Introduction

With the increased pace of life and unhealthy lifestyles, the prevalence of mental health conditions and substance abuse has risen rapidly in recent years [1]. Moreover, the capacities of health service systems in many countries are lagging, and people’s needs for psychological services are not met in a timely manner [2]. In the first year of the coronavirus disease 2019 (COVID-19) pandemic, the incidence of common psychiatric conditions such as depression and anxiety increased by 25% worldwide [3]. An epidemiological survey conducted in China showed that the weighted lifetime prevalence of psychiatric disorders (except dementia) among the population over 18 years of age is 16.57%; however, in early 1980, the prevalence of psychiatric disorders in the population aged over 15 years was only 12.69% [4]. Data from the United Kingdom Household Longitudinal Study (UKHLS) showed that the population prevalence of mental health issues increased from 24.3% between 2017 and 2019 to 37.8% in April 2020, with the largest increase (18.6%) among individuals aged 18–34 years [5]. Since the peak of the COVID-19 pandemic, mental health issues have remained prominent as people return to the workplace after a prolonged period of ‘working from home’. The burden of mental health issues not only increases the cost of medical treatment but also reduces human capital and productivity [6].

The nature of healthcare work is professional with a focus on social humanity. Healthcare professionals (HCPs) are involved in diagnosis and treatment and deal with complex interpersonal relationships with patients and their families. In addition, high social expectations, heavy workloads, excessive evaluations, high competition, and tension in relationships with colleagues and leadership may cause psychological strain among healthcare professionals [7]. However, healthcare institutions often pay little attention to the psychological health of staff and may not provide adequate support and resources [8]. A study from Germany showed that the COVID-19 pandemic caused symptoms of hypertonia, depression, and anxiety among healthcare professionals, and these symptoms tended to occur simultaneously [9]. A systematic review including 13 studies reported that the prevalence rates of anxiety, depression, and insomnia among healthcare professionals were 23.2%, 22.8%, and 38.9%, respectively [10]. Sevliya et al. found that during the normalization stage of the COVID-19 pandemic, mental health issues remained prominent among pediatricians, and the prevalence rates of depression, anxiety, and stress symptoms were 54.8%, 49.4%, and 33.3%, respectively [11]. An online survey conducted by the British Medical Association revealed that as doctors work longer hours, they are more likely to experience emotional distress [12]. Another study showed that burnout is more pronounced among doctors who are younger, work more than 40 h per week, and work in tertiary hospitals [13]. It has also been shown that the underreporting of psychological illness, self-stigma, and concerns about confidentiality among medical staff can significantly hinder them from seeking help, thus leading to a poorer state of health [14]. Poor mental health in healthcare professionals will not only have an impact on their quality of life but also interfere with their daily clinical judgment and may eventually lead to a decline in the quality of the medical services they provide [15].

With the development of information technology, electronic resources are playing an increasingly important role in public health, and electronic health has emerged as a new medical model. The World Health Organization defines electronic health as a service that transforms health resources and care through electronic technology [16]. Electronic mental health (e-mental health) approaches (“mental health services and information delivered or enhanced through the internet and related technologies”) are receiving increasing attention and have been indicated to play a critical role in health management [17]. During the COVID-19 pandemic, e-mental health interventions became the preferred choice due to social distancing constraints [18]. Some scholars believe that e-mental health, owing to the advantage of eliminating spatial distance constraints, can provide essential services to people living in rural areas, greatly improving the accessibility and equity of health services [19]. Moreover, e-mental health is cost-effective and reduces financial burdens [20]. Some studies also showed that e-mental health interventions reduced help-seeking barriers, such as shame, stigma, and fear of exposure, due to anonymity [21].

However, existing research on the effectiveness of e-mental health interventions is contradictory [22]. Christensen et al.concluded that elderly individuals with depression found video counseling beneficial and supportive of their mental health [23]. Similarly, another systematic review found that e-mental health services effectively ameliorated stress, depression, burnout, insomnia, and alcohol abuse, with effectiveness potentially correlating with personal characteristics such as occupation type [24]. However, not all researchers agree with these findings. Considering the diversity of programs and variability of outcomes, one study asserted that the evidence supporting internet interventions for bipolar disorder remains limited [25]. Some studies have argued that many patients are resistant to online therapy because of concerns about the confidentiality of their medical data [26]. Another study that targeted male workers at high risk of depression concluded that e-mental health courses were effective in improving sleep and reducing stress, but the lack of personal relevance and interaction was a hindering factor [27]. Bolier et al. pointed out that there was no significant difference between online and offline interventions in improving the well-being of healthcare professionals, and the latter had very low acceptance and adherence rates. Research from Poland noted that e-mental health interventions had limited effectiveness in improving burnout and depression in healthcare workers, whether immediately postintervention or at follow-up [28]. Marshall et al. noted that much of the existing research has been conducted by program companies, and more empirical evidence is needed regarding the safety and effectiveness of e-mental health services [29].

Most of the previous research has focused on the entire population, adolescents, older adults, or patients with psychological disorders, and few studies have specifically paid attention to the effects of e-mental health interventions on healthcare professionals [30]. In this systematic review and meta-analysis, the three most common psychological symptoms (stress, anxiety, and depression symptoms) were selected as outcomes to determine the effectiveness of e-mental health services. With the combination of subgroup analysis and meta-regression, we further determined the most effective intervention method. The findings can provide knowledge for psychological interventions and evidence for promoting the well-being of healthcare personnel.

## Methods

In accordance with the Preferred Reporting Items for Systematic Reviews and Meta-Analyses (PRISMA) statement, a review protocol was first developed and then registered in the PROSPERO database at the Center for Reviews and Dissemination in the United Kingdom (CRD42022360906) [31].

### Search strategies

A three-step extensive search was performed according to the procedures proposed by the Cochrane Handbook for Systematic Reviews of Interventions [32]. First, we searched PubMed, Embase, Cochrane Library, PsycINFO, Web of Science, ProQuest Dissertations and Theses, China National Knowledge Infrastructure (CNKI), and Wanfang electronic databases from inception to April 13, 2022, to identify eligible trials and performed a second search on March 10, 2023. Second, a search was conducted for unpublished trials from a variety of clinical trial registries. Finally, the reference lists of similar systematic reviews and the included studies, grey literature, and target journals were hand-searched to maximize the number of potential trials.

Specific index terms and keywords were developed in line with the syntax rules of the databases to identify all relevant studies. Medical subject heading (MeSH) terms or database subject headings, including ‘health personnel’, ‘doctor’, ‘anxiety disorders’, ‘occupational stress’, ‘e-mental health’, ‘e-therapy’, ‘mental health services’, and ‘health education’, along with truncated and expanded keywords, were used to identify all available relevant studies. The detailed search strategy used for the eight databases is reported in the Supplementary material (Table S2).

There was no language filter applied during the identification of the studies to ensure that all relevant studies were identified [33]. Studies with titles or abstracts that met the selection criteria were selected for full-text evaluation by two independent reviewers, and the reasons for exclusion were documented. A third author was involved in the resolution of any disagreements [34]. All relevant abstracts from the databases were uploaded into EndNote X8. The two reviewers independently removed duplicated abstracts and then reviewed the remaining abstracts for inclusion. Original authors of relevant trials were contacted via e-mail to request missing information or to seek clarification.

### Study eligibility criteria

Eligibility criteria were defined using the Population, Intervention, Comparison, and Outcome (PICO) framework. Studies qualified if they (1) involved healthcare professionals; (2) focused on e-mental health interventions; (3) included a control group with no treatment, usual care, or an active intervention; (4) measured at least one of the following outcomes: stress, depression, or anxiety; and (5) were randomized controlled trials (RCTs). Studies were excluded if they were (1) non-experimental studies; (2) discussion or review papers; (3) trial protocols; or (4) only abstracts. The specific eligibility criteria are reported in the Supplementary material (Table S3).

### Study selection

Two researchers (ZZM and YJH) screened the literature independently. Initial screening was performed by reading the titles and abstracts according to the inclusion and exclusion criteria. Then, for the remaining literature, a thorough full-text reading and further screening were conducted. Controversies were resolved by consensus of the two reviewers and were finalized by a third reviewer (VXW).

### Data extraction

A data extraction form for randomized trials was downloaded from the *Cochrane Handbook for Systematic Reviews of Interventions,* and data extraction was performed independently by two researchers [35]. The extracted information included the author, publication year, country, setting, research design, targeted population, age, sample size, intervention, follow-up, comparator, outputs, attrition rate, intention-to-treat (ITT), and missing data management. Information about e-mental health interventions, including the name, content, duration, follow-up, and outcome measures, was also extracted. Two researchers completed data extraction and then checked for differences, which were finalized by a third researcher.

### Quality assessment

The Cochrane risk-of-bias tool was used for bias evaluation [32]. Two researchers independently assessed the risk of bias for the included RCTs, including seven items: random sequence generation, allocation concealment, blinding of participants and personnel, blinding of outcome assessment, incomplete outcome data, selective outcome reporting, and other biases. Each item score corresponds to one of three levels: high risk, low risk, and unclear risk.

The Grading of Recommendations, Assessment, Development, and Evaluation (GRADE) framework was used to evaluate the overall quality of the literature [36]. The evaluation was conducted considering the following factors: risk of bias, inconsistency, indirectness, imprecision, and other considerations. Each factor corresponds to one of three levels: not serious, serious, and very serious. The differences were assessed by a third researcher and agreed upon before being finalized.

### Statistical analysis

Data were collated and analyzed using RevMan 5.4.1 statistical software [37]. As psychological states involve continuous data measured on different scales, the standardized mean difference (SMD) was chosen as the effect indicator for analysis. All summary analyses were based on the DerSimonian and Laird random-effects model and inverse-variance method. When a random-effects model is used, the weights of each study are more similar, and the confidence interval for the result is wider. However, when the conditions of each study are different, the studies are more reflective of the real world [38]. If a study only provided the size of the sample and the data of the quartile, then the previous study was referred to estimate the mean and standard deviation [39, 40]. If a study did not provide enough data for meta-analysis, the corresponding authors were contacted by e-mail. If there was no response from the corresponding authors, descriptive statistics of the data were used.

Cochran’s *Q* (chi-square test) and *I*^2^ statistics were used to assess the heterogeneity of the included studies. For the significance level of the chi-square test, *p* < 0.05 indicated significant statistical heterogeneity. The *I*^2^ value provided information on the magnitude of heterogeneity, with values below 40% considered not important, 30% to 60% indicating moderate heterogeneity, 50 to 90% indicating severe heterogeneity, and 75 to 100% indicating significant heterogeneity [35].

Sensitivity, subgroup, and meta-regression analyses were performed when the heterogeneity was significant. Sensitivity analysis involved systematically excluding one study at a time to assess its impact on the effect size and heterogeneity [32]. Subgroup analysis was conducted to explore the sources of heterogeneity and examine the differences in the effectiveness of e-mental health interventions across different groups [41]. Based on the literature, we identified the types of subgroups [34, 42]. Predefined subgroups included the publication year, research design, occupation type, intervention context, intervention tool, intervention technology, intervention approach, supportive system, and comparators. Meta-regression was performed using Stata 16.1 software to determine whether the heterogeneity between studies was influenced by specific covariates [38]. Random-effect univariate regression models were used to assess the effect of the sample size and duration of the intervention on the effectiveness of the intervention among healthcare providers. We adopted a significance level of *p* < 0.05 [43].

Publication bias was assessed when ten or more studies were included [44]. To examine publication bias, contour-enhanced funnel plots were used to calculate the comparison between each effect size and standard deviation. We also used the graphical test method proposed by Egger et al. to visually evaluate the funnel plot and determine the impact of publication bias on the interpretation of the results [43].

## Results

### Search results

**Fig. 1:**
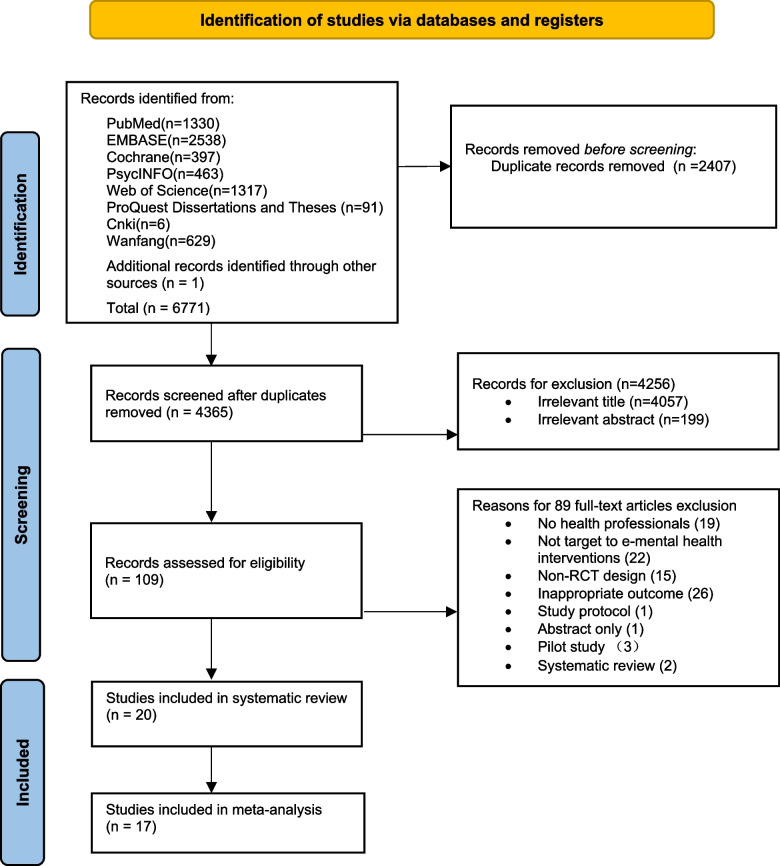
PRISMA flow diagram

A total of 6771 articles were obtained from searches of the eight databases combined with manual searches. After eliminating duplicates, 4365 articles were further screened. After reading the titles and abstracts, 4265 articles were excluded. Eighty-nine articles were further excluded after reading the full text. Ultimately, 20 articles were included in the systematic review. Due to missing data or differences in analysis methods in some articles, we only conducted a meta-analysis on 17 articles. The literature screening process and studies are shown in Fig. 1.

### Study characteristics

Of the 20 studies included, seven were from China [45–51], five were from the USA [52–56], and the rest were from Australia [57], Germany [58], Ireland [59], the Netherlands [60], Poland [28], Spain [61], Turkey [62], and Vietnam [63]. There were fifteen RCTs with two arms, four RCTs with three arms, and one RCT with four arms. The sample size ranged from 36 to 1240 participants. Eleven studies targeted nurses, one targeted doctors, and eight targeted mixed populations. The psychological interventions included mindfulness-based therapy, cognitive–behavioral therapy (CBT), stress management, emotional freedom techniques (EFTs), and mixed techniques. Fourteen studies used individual interventions, and six studies applied group interventions. The intervention duration ranged from ten days to half a year. The highest dropout rate was 82.15%. In addition to stress, anxiety, and depression symptoms, some studies also included burnout, well-being, work engagement, and self-efficacy as outcome variables. The characteristics and interventions of each study are shown in the Supplementary material (Tables S5 and S6).

### Risk of *bias* within studies

Among the included studies, only two were classified as having a low risk of bias for each item [57, 61]. Two studies were classified as having a high risk of bias for randomization [55, 56]. Most studies did not clarify the ways in which allocation concealment or participant, researcher, or data processor blinding was implemented [28, 45–56, 58–60, 63]. Seven studies were classified as having a high risk of bias for incomplete data reporting due to high rates of participant dropout and a lack of explanation of the causes [28, 47, 53–56, 60]. A detailed quality assessment of the studies is shown in Supplementary material (Fig. S1).

### Risk of *bias* across studies

According to the GRADE criteria, the overall quality of the studies related to the three outcome indicators was evaluated as low (see Supplementary Material Table S7). For stress and anxiety symptoms, the risk of inconsistency was downgraded due to the very large heterogeneity. For depression symptoms, the risk of bias was downgraded, as allocation concealment and blinding were not well implemented. A funnel plot was used to analyze publication bias regarding stress and anxiety symptoms. The funnel plot was symmetrical, and no study accepted sponsorship from stakeholders, which suggests that there was no obvious publication bias.

## Results of the *meta*-analysis

**Fig. 2:**
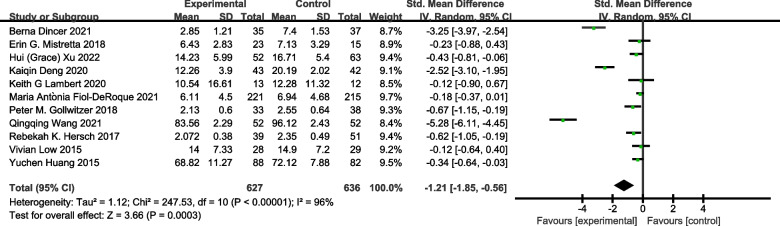
Forest plot of the effect of e-mental health on stress

**Fig. 3:**
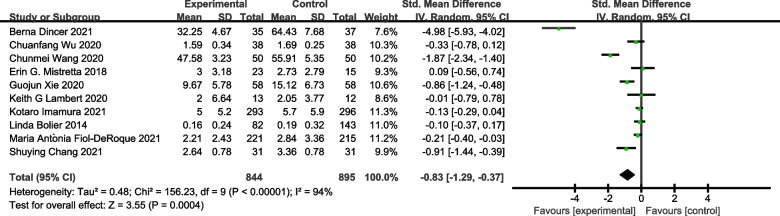
Forest plot of the effect of e-mental health on anxiety

**Fig. 4:**
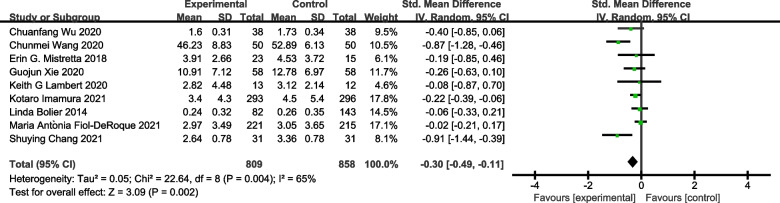
Forest plot of the effect of e-mental health on depression

Eleven studies including 1263 subjects were included in the meta-analysis on the outcome measure of stress. As shown in Fig. 2, stress symptoms were significantly improved in the e-mental health intervention group compared with the control group [SMD = − 1.21, 95% CI (− 1.85, − 0.56), *P* < 0.05]. Ten studies were included in the meta-analysis on symptoms of anxiety. As shown in Fig. 3, anxiety symptoms were significantly improved in the e-mental health intervention group compared with the control group [SMD = − 0.83, 95% CI (− 1.29, − 0.37), *P* < 0.05]. As shown in Fig. 4, from the meta-analysis of the remaining nine studies, depression was moderately reduced in the intervention group [SMD = − 0.30, 95% CI (− 0.49, − 0.11),* P* < 0.05]. Due to the significant heterogeneity [stress symptoms: *I*^2^ = 96%, *P* < 0.05; anxiety: *I*^2^ = 94%, *P* < 0.05; depression symptoms: *I*^2^ = 65%, P < 0.05], we conducted a sensitivity analysis. After removing the studies one by one, the meta-analysis was not affected, indicating that the results were stable.

### Subgroup analysis

For stress symptoms, significant differences were noted in the effect of the interventions among the following subgroups: publication year, design, occupation type, intervention tool, intervention technology, intervention approach, supportive system, and control group. For anxiety symptoms, significant differences were observed among the publication year, occupation type, intervention context, intervention tool, intervention technology, intervention approach, and supportive system subgroups. For depression symptoms, there were significant differences in the effects among the region, occupation type, intervention technology, intervention approach, and supportive system subgroups. This partly explained the sources of heterogeneity. Notably, certain subgroups had a few studies or exhibited high heterogeneity, thus warranting caution in the generalizability of our results. The detailed data are shown in Table 1.

**Table 1:** Subgroup analysis of e-mental health intervention on stress, anxiety, and depression among healthcare professionals

Subgroups	Stress						Anxiety						Depression					
	Number of studies	Sample size	*I*^2^ (%)	SMD 95%CI	Overall effect (*Z* value)	Subgroup differences (Chi2 value)	Number of studies	Sample size	*I*^2^ (%)	SMD 95%CI	Overall effect (*Z* value)	Subgroup differences (Chi2 value)	Number of studies	Sample size	*I*^2^ (%)	SMD 95%CI	Overall effect (*Z* value)	Subgroup differences (Chi2 value)
Publication year																		
≥ 2020	6	837	98	− 1.94 [− 3.25,− 0.63]	2.90*	5.13*	8	1476	95	− 1.06 [− 1.62, − 0.49]	3.65*	9.63*	7	1404	72	− 0.36 [− 0.60, − 0.13]	3.02*	2.64
< 2020	5	426	0	− 0.41 [− 0.60,− 0.22]	4.14*		2	263	0	− 0.07 [− 0.32, 0.18]	0.58		2	263	0	− 0.08 [− 0.33, 0.17]	0.62	
Region																		
East	3	213	98	− 2.04 [− 4.73, 0.65]	1.49	0.65	5	943	93	− 0.80 [− 1.42, − 0.19]	2.56*	0.05	5	943	69	− 0.49 [− 0.78, − 0.20]	3.33*	7.26*
West	8	1050	94	− 0.91 [− 1.50, − 0.33]	3.06*		5	796	96	− 0.93 [− 1.83, − 0.04]	2.05*		4	724	0	− 0.04 [− 0.19, 0.10]	0.58	
Design																		
2-arm	9	1154	97	− 1.38 [− 2.15, − 0.61]	3.52*	3.85*	7	996	96	− 1.11 [− 1.84, − 0.39]	3.02*	2.95	6	924	78	− 0.37 [− 0.69, − 0.05]	2.25*	0.61
> 2-arm	2	109	12	− 0.51 [− 0.92, − 0.09]	2.38*		3	743	85	− 0.32 [− 0.87, 0.22]	1.17		3	743	0	− 0.23 [− 0.37, − 0.09]	3.11*	
Job																		
Nurse	6	592	97	− 2.08 [− 3.32, − 0.83]	3.26*	8.41*	6	890	91	− 0.54 [− 1.13, 0.04]	1.81	2.79*	5	943	69	− 0.49 [− 0.78, − 0.20]	3.33*	7.26*
All	5	671	0	− 0.22 [− 0.37, − 0.07]	2.81*		4	849	97	− 1.36 [− 2.32, − 0.41]	2.79*		4	724	0	− 0.04 [− 0.19, 0.10]	0.58	
Intervention context																		
CBT	3	298	17	− 0.93 [− 1.68, − 0.19]	2.81*	2.00	1	116	_	− 0.86 [− 1.24, − 0.48]	4.44*	107.08*	1	116	_	− 0.26 [− 0.63, 0.10]	1.41	3.39
Mindfulness	4	282	98	− 1.50 [− 3.49, 0.49]	1.48		4	239	91	− 0.55 [− 1.52, 0.41]	1.12		4	239	43	− 0.47 [− 0.83, − 0.11]	2.53*	
Stress management	1	90	_	− 0.62 [− 1.05, − 0.19]	2.84*		_						_					
EFT	_						1	72	_	− 4.98 [− 5.93, − 4.02]	10.21*		_					
Mixed	_						3	1250	0	− 0.15 [− 0.26, − 0.04]	2.68*		3	1250	30	− 0.12 [− 0.25, 0.02]	1.69	
Intervention tool																		
Computer	4	332	97	− 2.91 [− 4.74, − 1.08]	3.12*	8.32*	5	575	97	− 1.66 [− 2.75, − 0.58]	3.01*	7.31*	4	503	80	− 0.49 [− 0.92, − 0.07]	2.27*	2.25
Phone	5	784	0	− 0.25 [− 0.39, − 0.11]	3.49*		5	1164	0	− 0.16 [− 0.28, − 0.05]	2.78*		5	1164	0	− 0.16 [− 0.27, − 0.04]	2.65*	
Mixed	2	147	52	− 0.39 [− 0.88, 0.09]	1.59		-						-					
Intervention technology																		
APP	6	841	0	− 0.24 [− 0.38, − 0.11]	3.49*	23.27*	5	1164	0	− 0.16 [− 0.28, − 0.05]	2.78*	7.13*	5	1164	0	− 0.16 [− 0.27, − 0.04]	2.65*	18.09*
Online course	3	261	93	− 3.66 [− 5.20, − 2.12]	4.67*		3	234	96	− 2.53 [− 4.35, − 0.72]	2.74*		2	162	0	− 0.89 [− 1.21, − 0.56]	5.37*	
Website	2	161	0	− 0.64 [− 0.96, − 0.32]	3.93*		2	341	90	− 0.47 [− 1.22, 0.28]	1.24		2	341	0	− 0.13 [− 0.35, 0.09]	1.18	
Intervention approach																		
Individual	7	832	16	− 0.32 [− 0.49, − 0.16]	3.82*	5.31*	7	1505	58	− 0.24 [− 0.43, − 0.06]	2.54*	6.07*	7	1505	0	− 0.15 [− 0.25, − 0.05]	2.89*	18.05*
Group	4	431	98	− 2.83 [− 4.95, − 0.70]	2.61*		3	234	96	− 2.53 [− 4.35, − 0.72]	2.74*		2	162	0	− 0.89 [− 1.21, − 0.56]	5.37*	
Supportive system																		
Self-guided	6	775	25	− 0.36 [− 0.55, − 0.17]	3.68*	5.03*	7	1505	58	− 0.24 [− 0.43, − 0.06]	2.54*	6.07*	7	1505	0	− 0.15 [− 0.25, − 0.05]	2.89*	18.05*
Professional support	5	488	98	− 2.28 [− 3.94, − 0.61]	2.68*		3	234	96	− 2.53 [− 4.35, − 0.72]	2.74*		2	162	0	− 0.89 [− 1.21, − 0.56]	5.37*	
Control group																		
Not active	10	827	96	− 1.33 [− 2.11, − 0.54]	3.31*	7.76*	8	1241	95	− 0.94 [− 1.57, − 0.31]	2.91*	0.80	7	1169	47	− 0.29 [− 0.48, − 0.10]	3.01*	0.09
Active	1	436	_	− 0.18 [− 0.37, − 0.01]	1.88		2	498	83	− 0.52 [− 1.20, 0.16]	1.50		2	498	90	− 0.43 [− 1.30, 0.44]	0.97	

*CI* confidence interval, *APP* application, *CBT* cognitive-behavioral therapy, *EFT* emotional freedom techniques, *SMD* standardized mean difference

^*^p < 0.05

In terms of publication year, articles published after 2020 had significantly higher intervention effects than those published before 2020 (stress symptoms: SMD (≥ 2020) = − 1.94, *Z* = 2.90, *P* < 0.05 versus SMD (< 2020) = − 0.41, *Z* = 4.14, *P* < 0.05; anxiety symptoms: SMD (≥ 2020) = − 1.06, *Z* = 3.65, *P* < 0.05 versus SMD (< 2020) = − 0.07, *Z* = 0.58, *P* > 0.05; and depression symptoms: SMD (≥ 2020) = − 0.36, *Z* = 3.02, *P* < 0.05 versus SMD (< 2020) = − 0.08, *Z* = 0.62, *P* > 0.05).

For intervention groups, more studies focused on nurses compared to other healthcare professionals, and the intervention effect was better. With regard to stress and depression symptoms, the intervention effect was significantly better for nurses than for all other healthcare staff (stress symptoms: SMD (nurses) = − 2.08, *Z* = 3.26, *P* < 0.05 versus SMD (all) = − 0.22, *Z* = 2.81, *P* < 0.05; depression symptoms: SMD (nurses) = − 0.49, *Z* = 3.33, *P* < 0.05 versus SMD (all) = − 0.04, *Z* = 0.58, *p* > 0.05), and the difference between the groups was significant (stress symptoms: *χ*2 = 8.41, *p* > 0.05; depression symptoms: *χ*2= 7.26, *p* > 0.05).

Regarding stress and depression symptoms, mindfulness therapy exhibited a better effect than CBT (stress symptoms: SMD (CBT) = − 0.93, *Z* = 2.81, *P* < 0.05 versus SMD (mindfulness) = − 1.50, *Z* = 1.48, *P* < 0.05; depression symptoms: SMD (CBT) = − 0.26, *Z* = 1.41, *p* > 0.05 versus SMD (mindfulness) = − 0.47, *Z* = 2.53, *P* < 0.05). However, there was no significant difference between the groups. For anxiety symptoms, EFTs showed a better therapeutic effect, followed by CBT and mindfulness therapy, and then mixed interventions, with significant differences between the groups.

Regarding intervention technology, online course interventions were significantly better than the other two methods, namely, the app and website methods, and there were significant differences between the groups (stress symptoms: SMD (app) = − 0.24 versus SMD (online course) = − 3.66 versus SMD (website) = − 0.64, *χ*2 = 23.27,* P* < 0.05; anxiety symptoms: SMD (app) = − 0.16 versus SMD (online course) = − 2.53 versus SMD (website) = − 0.47, *χ*2=7.13, *P* < 0.05; depression symptoms: SMD (app) = − 0.16 versus SMD (online course) = − 0.89 versus SMD (website) = − 0.13, *χ*2=18.09, *P* < 0.05).

Group interventions seemed to be more effective than individual interventions (stress symptoms: SMD = − 2.83, 95% CI [− 4.95, − 0.70], *Z* = 2.61, *P* < 0.05 for group versus SMD = − 0.32, 95% CI [− 0.49, − 0.16], *Z* = 3.82, *P* < 0.05 for individual; anxiety symptoms: SMD = − 2.53, 95% CI [− 4.35, − 0.72], *Z* = 2.74, *P* < 0.05 for group versus SMD = − 0.24, 95% CI [− 0.43, − 0.06], *Z* = 2.54, *P* < 0.05 for individual; depression symptoms: SMD = 0.89, 95% CI [− 1.21, − 0.56], *Z* = 5.37, *P* < 0.05 for group versus SMD = − 0.15, 95% CI [− 0.25, − 0.05], *Z* = 2.89, *P* < 0.05 for individual). Significant differences were found within a specific subgroup (stress symptoms: *χ*2 = 5.31, *p* < 0.05; anxiety: *χ*2 = 6.07, *p* < 0.05; depression symptoms: *χ*2 = 18.05, *p* < 0.05), indicating that the intervention effect size varied significantly within this subgroup.

Professional support interventions were more effective than self-guided interventions (stress symptoms: SMD (professional support) = 2.28, 95% CI [− 3.94, − 0.61], *Z* = 2.68, *P* < 0.05 versus SMD (self-guided interventions) = − 0.36, 95% CI [− 0.55, − 0.17], *Z* = 3.68, *P* < 0.05; anxiety symptoms: SMD (professional support) = − 2.53, 95% CI [− 4.35, − 0.72], *Z* = 2.74, *P* < 0.05 versus SMD (self-guided interventions) = − 0.24, 95% CI [− 0.43, − 0.06], *Z* = 2.54, *P* < 0.05; depression symptoms: SMD (professional support) = − 0.89, 95% CI [− 1.21, − 0.56], *Z* = 5.37, *P* < 0.05 versus SMD (self-guided interventions) =− 0.15, 95% CI [− 0.25, − 0.05], *Z* = 2.89, *P* < 0.05). Significant differences were found within a specific subgroup (stress symptoms: *χ*2 = 5.03, *p* < 0.05; anxiety: *χ*2 = 6.07, *p* < 0.05; depression symptoms: *χ*2= 18.05,* p* < 0.05), indicating that the intervention effect size varied significantly within this subgroup.

### *Meta*-regression

Meta-regression demonstrated that the intervention duration (β = 0.027, *P* = 0.04) had a significant effect on the relief of anxiety symptoms. For stress and depressive symptoms, the intervention duration and sample size had no significant impact. Detailed results are displayed in Table 2.

**Table 2:** Random effects meta-regression models of e-mental intervention on stress, anxiety, and depression

Covariate	Stress					Anxiety					Depression				
	β	SE	95%CI	*z*	*P* value	β	SE	95%CI	z	*P* value	β	SE	95%CI	*z*	*P* value
Intervention duration	0.011	0.011	− 0.110, 0.033	1.010	0.310	0.027	0.013	0.001, 0.054	2.050	0.040*	0.005	0.004	− 0.003, 0.012	1.200	0.229
Sample size	0.004	0.005	− 0.006, 0.014	0.770	0.441	0.002	0.002	− 0.003, 0.006	0.760	0.450	0.001	0.001	− 0.0004, 0.002	1.270	0.204
_cons	− 2.272	1.083	− 4.394, 0.149	− 2.10	0.036	− 2.491	0.816	− 4.090, − 0.891	− 3.050	0.002	− 0.704	0.249	− 1.193, − 0.216	− 2.820	0.005

^*^p < 0.05, *β* = regression coefficient, *z* = *z* statistics

### Other outcomes

Other outcomes in the studies included burnout, well-being, work engagement, self-efficacy, satisfaction, attention, somatic symptoms, sleep quality, trauma exposure, compassion, social support, work-life integration, and coping style, among which burnout and well-being were most commonly included, with five studies each. From the results shown in Table 3, e-mental health interventions significantly alleviated emotional exhaustion [SMD = − 0.91, 95% CI (− 1.33, − 0.50), *P* < 0.05] and improved the well-being of subjects [SMD = 0.38, 95% CI (0.01, 0.75), *P* < 0.05] but had no impact on depersonalization and personal accomplishment.

**Table 3:** Effectiveness of e-mental health intervention on secondary outcomes among medical staff

Outcomes	Number of trials	Sample size	*I*^*2*^(%)	SMD 95%CI	Overall effect (*Z* value)
Burnout Emotional exhaustion	5	686	0	− 0.91 [− 1.33, − 0.50]	4.28*
Burnout depersonalization	5	686	71	− 0.16 [− 1.38, 1.07]	0.25
Burnout personal accomplishment	5	686	71	0.34 [− 1.16, 1.85]	0.45
Well-being	5	730	83	0.38 [0.01, 0.75]	2.01*

^*^p < 0.05

## Discussion

To our knowledge, this review appears to be among the first to provide a systematic and comprehensive analysis of the impact of e-mental health interventions on the well-being of healthcare staff in a professional context [53]. Our results were derived from 17 RCTs, totaling 2431 subjects. Compared with the control groups, the e-mental health intervention groups showed significant effects on stress, anxiety, and depression symptoms. Subgroup analysis demonstrated a series of moderators, including intervention context and supportive system.

E-mental health interventions exhibited large effects in mitigating stress and anxiety symptoms among healthcare professionals and showed small-to-moderate effects in reducing depression symptoms, which follows the general trends reported in related studies. In another review, a significant effect (Hedge’s *g* = 0.51, *P* < 0.05) that was lower than that in our study was observed in the reduction in stress symptoms in the general population [64]. This may be due to the higher health literacy of healthcare staff, so more obvious effects were observed after the interventions. Another meta-analysis showed a significant effect (Cohen’s *d* = − 0.91, *P* < 0.05), which is comparable to our results for generalized anxiety disorders [65]. Similar to our findings, e-mental health interventions were determined to have a significant effect on depression symptoms (Hedge’s *g* = 0.30, *P* < 0.05) based on 17 studies in another meta-analysis [24]. Excessive workloads, prolonged overtime hours, competitive coworker relationships, and the occurrence of patient death have blurred work-life boundaries and negatively impacted the psychological well-being of healthcare workers [65]. Additionally, healthcare professionals usually have busy work schedules and lack time to attend face-to-face psychological sessions. E-mental health interventions provide a channel for healthcare professionals to focus on their own psychological health. Specifically, e-mental health services are characterized by eliminating time and space constraints to improve accessibility and compliance [66]. By acquiring relaxation skills and emotional support, healthcare professionals can alleviate inner discomfort and adjust their cognitive thinking, which in turn can help them communicate well with others and improve their quality of life [67]. In light of the existing research, e-mental health interventions can be complementary or alternatives to face-to-face interventions for healthcare professionals.

The results also supported the positive effects of e-mental health interventions for some secondary outcomes, indicating that the research had good systematic consistency. In terms of well-being, the intervention effect we obtained had an SMD = 0.38 (95% CI 0.01‒0.75, *P* < 0.05). Phillips et al. reported an effect size on well-being of Hedge’s *g* = 0.35 (95% CI 0.25‒0.46, *P* < 0.0001) based on seven studies [24]. For burnout, a significant effect of the intervention was observed in reducing emotional exhaustion (SMD = − 0.91, *Z* = 4.28, *P* < 0.05), but no significant effect was observed in reducing depersonalization and establishing a sense of achievement. Another study found that online interventions had moderate-to-large effects on job burnout [68]. These conflicting conclusions may exist because we only considered burnout as a secondary outcome and only included a small number of studies.

The subgroup analysis showed that mindfulness meditation techniques had a higher effect size than other psychological techniques, such as CBT. However, this result should be interpreted with caution, as the sample size of each subgroup was small, and the heterogeneity was high. Mindfulness refers to a state of consciousness created by directing attention to the present goal and indulging in the moment without distractions or judgment [69]. As a lifestyle-based intervention, mindfulness is cost-effective and has no side effects [70]. In addition to stress and depression, researchers have found that it can also alleviate other physiological problems, such as PTSD, pain, and eating disorders [71]. In the future, other psychological interventions, such as acceptance and commitment therapy, life-review therapy, and dialectical behavior therapy, could be integrated into e-mental health interventions to further explore their effectiveness in improving psychological health [72]. Our results also indicated that computer-based online interventions were more effective than mobile apps. However, the applicability of computer-based interventions may be potentially limited by the high heterogeneity of the corresponding subgroups, and medical personnel who attend online courses may also use their mobile phones to browse health information in their daily lives. Our explanatory subgroup analyses verified the advantage of professional support and group interventions. Previous reviews have revealed that guided psychotherapy is more effective than unguided psychotherapy owing to external pressure fostering individual accountability and positive reinforcement [73]. Baumeister and colleagues reported that internet-based mental health interventions (IMIs) together with therapeutic support had a significantly lower dropout rate (odds ratio = 2.67) and achieved a greater reduction in symptoms (Hedge’s *g* = − 0.27, *P* < 0.05) than IMIs without such support [74]. Some studies have suggested that group interventions can enhance mutual interaction and trust, facilitate emotional expression, and thus improve effectiveness [73]. However, other studies have reported that group interventions are less effective than individual delivery due to inadequate attention for each individual [75]. Therefore, it is necessary to further explore customized interventions based on the personal characteristics and job requirements of healthcare personnel to ensure program effectiveness.

According to the meta-regression, the intervention duration had a significant slight impact on anxiety symptoms (β = 0.027, *p* = 0.040) but no impact on the relief of stress and depression symptoms, suggesting that the design of the program should be based on clinical symptoms and that short-term interventions are more cost-effective [76]. It is also worth noting that this result could potentially be attributed to chance, as the value of 0.04 is truly at the threshold. From the meta-regression, the number of people included in the RCTs did not affect the final outcome. From the subgroup analysis, no significant difference was found with different types of control groups. In the subgroup analysis, no significant difference was found when comparing non-active control groups (no treatment, waiting list, treatment-as-usual) and active control groups (placebos such as general recommendations about mental health care, or relaxation sessions) with the intervention groups. Of course, more research on various intervention forms (e-package, social media platforms, virtual care, online platforms, online games) is needed to confirm this.

### Strengths and limitations

We performed an extensive and systematic literature review, including references to related reviews and RCTs. Then, we further performed subgroup analysis and meta-regression to identify the relative effect size of various moderators, which provides guidance for future research and practical design. Notably, publication biases were not detected in the studies we included. Moreover, in addition to using the risk of bias tool to evaluate the quality of individual trials, we also used the GRADE criteria to evaluate the overall quality of evidence.

Several limitations should be considered before interpreting the findings. Firstly, the included trials exhibited clinical and statistical heterogeneity, which limited their comparability. To address this issue, we conducted a subgroup analysis to explore potential sources of heterogeneity. Moderators that emerged consistently across the four main outcomes—job, intervention technology, intervention approach, and supportive system—could explain the observed heterogeneity. Secondly, we found that the overall quality of evidence was deemed low, primarily due to issues with allocation concealment, blinding, and missing outcome data. These issues may have compromised the internal validity of our findings. Lastly, it is important to note that the outcomes of this review were primarily based on self-reported data, which could introduce recall and/or social desirability bias. Future trials may benefit from using established diagnostic criteria manuals, such as the DSM-5 (Diagnostic and Statistical Manual of Mental Disorders) or ICD-10 (International Classification of Diseases).

### Implications for future research and practice

Given that the number of RCTs included was not very large, our systematic review suggested that more attention should be given to the psychological health of healthcare staff. In addition, larger sample size, multicenter, longitudinal, or follow-up studies are needed. Considering the low quality of the studies, rigorous RCTs following the CONSORT statement are recommended for future trials, especially efforts to minimize selection bias by performing adequate allocation concealment and reducing performance bias by blinding participants and researchers. Addressing emerging issues such as inequalities caused by the digital divide, the potential disclosure of privacy, and the lack of user trust is necessary. Future research could consider using objective outcomes such as biological markers or more sophisticated and intensive e-mental health approaches [77]. Based on the principles of user-centered designs and incorporating feedback from users and providers, e-mental health interventions also require codesigning and integrating cross-disciplinary collaboration among psychology, health science, and computer professionals [78].

## Conclusion

Mental health interventions were found to significantly alleviate stress, anxiety, depression symptoms, and other emotional issues among healthcare professionals. Subgroup analyses showed that features including mindfulness approaches, online courses, computer use, group interventions, and professional guidance are more favorable in the design of services. Meta-regression showed that the intervention duration, as a covariate, only had an impact on preventing or reducing symptoms of anxiety, while the sample size did not affect any of the three primary symptoms. Our findings on efficacy outcomes should be interpreted cautiously due to the high heterogeneity observed in some analyses and the GRADE approach indicating a low quality of evidence. E-mental health interventions have the potential to become widely available and evidence-based interventions to address mental health issues. Future RCTs should adhere to the recommendations of the CONSORT statement, as more high-quality trials are warranted to assess the effectiveness of e-mental health interventions among healthcare professionals.

### Supplementary Information

Additional file 1: Supplementary Table S1. PRISMA checklist. Supplementary Table S2. Search terms of databases. Supplementary Table S3. Eligibility criteria. Supplementary Table S4. Data extraction table. Supplementary Table S5. Characteristics of included studies. Supplementary Table S6. Description of e-mental health intervention. Supplementary Table S7. Summary of Findings table. Supplementary Figure S1. Risk of bias within studies summary. Supplementary Figure S2. Funnel plot of stress. Supplementary Figure S3. Funnel plot of anxiety.

## Data Availability

Supplemental material for this paper is available online. Other information will be made available upon request to the author.
