# Supplementary material for: The effectiveness of e-mental health interventions on stress, anxiety, and depression among healthcare professionals: a systematic review and meta-analysis
Source: Syst Rev. 2024 May 30;13:144. doi: 10.1186/s13643-024-02565-6 (PMC11138032; doi:10.1186/s13643-024-02565-6)
Supplement: Supplementary file 1 — Additional file 1: Supplementary Table S1. PRISMA checklist. Supplementary Table S2. Search terms of databases. Supplementary Table S3. Eligibility criteria. Supplementary Table S4. Data extraction table. Supplementary Table S5. Characteristics of included studies. Supplementary Table S6. Description of e-mental health intervention. Supplementary Table S7. Summary of Findings table. Supplementary Figure S1. Risk of bias within studies summary. Supplementary Figure S2. Funnel plot of stress. Supplementary Figure S3. Funnel plot of anxiety. [file 13643_2024_2565_MOESM1_ESM.docx]

**Supplementary material**

## Table 1: PRISMA checklist

| **Section and Topic** | **Item #** | **Checklist item** | **Location where item is reported** |
| --- | --- | --- | --- |
| **TITLE** | | |  |
| Title | 1 | Identify the report as a systematic review. | 1 |
| **ABSTRACT** | | |  |
| Abstract | 2 | See the PRISMA 2020 for Abstracts checklist. | 2 |
| **INTRODUCTION** | | |  |
| Rationale | 3 | Describe the rationale for the review in the context of existing knowledge. | 2-4 |
| Objectives | 4 | Provide an explicit statement of the objective(s) or question(s) the review addresses. | 4 |
| **METHODS** | | |  |
| Eligibility criteria | 5 | Specify the inclusion and exclusion criteria for the review and how studies were grouped for the syntheses. | 4-5,  Supplementary Table 3 |
| Information sources | 6 | Specify all databases, registers, websites, organisations, reference lists and other sources searched or consulted to identify studies. Specify the date when each source was last searched or consulted. | 4-5, Figure 1 |
| Search strategy | 7 | Present the full search strategies for all databases, registers and websites, including any filters and limits used. | 4-5, Supplementary Table 2 |
| Selection process | 8 | Specify the methods used to decide whether a study met the inclusion criteria of the review, including how many reviewers screened each record and each report retrieved, whether they worked independently, and if applicable, details of automation tools used in the process. | 5-6 |
| Data collection process | 9 | Specify the methods used to collect data from reports, including how many reviewers collected data from each report, whether they worked independently, any processes for obtaining or confirming data from study investigators, and if applicable, details of automation tools used in the process. | 5-6 |
| Data items | 10a | List and define all outcomes for which data were sought. Specify whether all results that were compatible with each outcome domain in each study were sought (e.g. for all measures, time points, analyses), and if not, the methods used to decide which results to collect. | 6-7, Supplementary Table 5-7 |
|  | 10b | List and define all other variables for which data were sought (e.g. participant and intervention characteristics, funding sources). Describe any assumptions made about any missing or unclear information. | Supplementary Table 5-7 |
| Study risk of bias assessment | 11 | Specify the methods used to assess risk of bias in the included studies, including details of the tool(s) used, how many reviewers assessed each study and whether they worked independently, and if applicable, details of automation tools used in the process. | 5-6 |
| Effect measures | 12 | Specify for each outcome the effect measure(s) (e.g. risk ratio, mean difference) used in the synthesis or presentation of results. | 6 |
| Synthesis methods | 13a | Describe the processes used to decide which studies were eligible for each synthesis (e.g. tabulating the study intervention characteristics and comparing against the planned groups for each synthesis (item #5)). | 6-7 |
|  | 13b | Describe any methods required to prepare the data for presentation or synthesis, such as handling of missing summary statistics, or data conversions. | 6-7 |
|  | 13c | Describe any methods used to tabulate or visually display results of individual studies and syntheses. | 6-7 |
|  | 13d | Describe any methods used to synthesize results and provide a rationale for the choice(s). If meta-analysis was performed, describe the model(s), method(s) to identify the presence and extent of statistical heterogeneity, and software package(s) used. | 6-7 |
|  | 13e | Describe any methods used to explore possible causes of heterogeneity among study results (e.g. subgroup analysis, meta-regression). | 6-7 |
|  | 13f | Describe any sensitivity analyses conducted to assess robustness of the synthesized results. | 6-7 |
| Reporting bias assessment | 14 | Describe any methods used to assess risk of bias due to missing results in a synthesis (arising from reporting biases). | 6-7 |
| Certainty assessment | 15 | Describe any methods used to assess certainty (or confidence) in the body of evidence for an outcome. | 6-7 |
| **RESULTS** | | |  |
| Study selection | 16a | Describe the results of the search and selection process, from the number of records identified in the search to the number of studies included in the review, ideally using a flow diagram. | 6-7  Figure 1 |
|  | 16b | Cite studies that might appear to meet the inclusion criteria, but which were excluded, and explain why they were excluded. | 6-7,  Figure 1 |
| Study characteristics | 17 | Cite each included study and present its characteristics. | 7-8,  Supplementary Table 5-6 |
| Risk of bias in studies | 18 | Present assessments of risk of bias for each included study. | 8, Supplementary Figure 1 |
| Results of individual studies | 19 | For all outcomes, present, for each study: (a) summary statistics for each group (where appropriate) and (b) an effect estimate and its precision (e.g. confidence/credible interval), ideally using structured tables or plots. | 8, Figure 2-4 |
| Results of syntheses | 20a | For each synthesis, briefly summarise the characteristics and risk of bias among contributing studies. | 8-9, Figure 2-4. Table 1-3 |
|  | 20b | Present results of all statistical syntheses conducted. If meta-analysis was done, present for each the summary estimate and its precision (e.g. confidence/credible interval) and measures of statistical heterogeneity. If comparing groups, describe the direction of the effect. | 8-9, Figure 2-4. Table 1-3 |
|  | 20c | Present results of all investigations of possible causes of heterogeneity among study results. | 8-9 Figure 2-4. Table 1-3 |
|  | 20d | Present results of all sensitivity analyses conducted to assess the robustness of the synthesized results. | 8 |
| Reporting biases | 21 | Present assessments of risk of bias due to missing results (arising from reporting biases) for each synthesis assessed. | 7-8, Supplementary Figure 2-3 |
| Certainty of evidence | 22 | Present assessments of certainty (or confidence) in the body of evidence for each outcome assessed. | 7-8 |
| **DISCUSSION** | | |  |
| Discussion | 23a | Provide a general interpretation of the results in the context of other evidence. | 10-12 |
|  | 23b | Discuss any limitations of the evidence included in the review. | 12-13 |
|  | 23c | Discuss any limitations of the review processes used. | 12-13 |
|  | 23d | Discuss implications of the results for practice, policy, and future research. | 13 |
| **OTHER INFORMATION** | | |  |
| Registration and protocol | 24a | Provide registration information for the review, including register name and registration number, or state that the review was not registered. | 4-5 |
|  | 24b | Indicate where the review protocol can be accessed, or state that a protocol was not prepared. | 4-5 |
|  | 24c | Describe and explain any amendments to information provided at registration or in the protocol. | N.A. |
| Support | 25 | Describe sources of financial or non-financial support for the review, and the role of the funders or sponsors in the review. | 13-1 |
| Competing interests | 26 | Declare any competing interests of review authors. | 13 |
| Availability of data, code and other materials | 27 | Report which of the following are publicly available and where they can be found: template data collection forms; data extracted from included studies; data used for all analyses; analytic code; any other materials used in the review. | 14 |

*From:*  Page MJ, McKenzie JE, Bossuyt PM, Boutron I, Hoffmann TC, Mulrow CD, et al. The PRISMA 2020 statement: an updated guideline for reporting systematic reviews. BMJ 2021;372:n71. doi: 10.1136/bmj.n71 For more information, visit: <http://www.prisma-statement.org/>

## Table 2: Search terms of databases

| **Database** | **Index terms and keywords** |
| --- | --- |
| **PubMed** | "Health Personnel"[MeSH Terms] OR “Medical staff”[MeSH Terms] OR "healthcare provider*"[Title/Abstract] OR "healthcare professional*"[Title/Abstract] OR "healthcare worker*"[Title/Abstract] OR "Allied Health"[Title/Abstract] OR "Dentist*"[Title/Abstract] OR "Paramedic*"[Title/Abstract] OR "Nurse*"[Title/Abstract] OR "Therapist*"[Title/Abstract] OR "Physician*"[Title/Abstract] OR "Doctor*"[Title/Abstract]  **AND**  Depression[MeSH] OR Anxiety[MeSH] OR "Anxiety disorders"[MeSH] OR "Stress, Psychological"[MeSH] OR "Occupational stress"[MeSH] OR "Depress*"[Title/Abstract] OR Anxiety[Title/Abstract] OR Stress[Title/Abstract] OR Burden[Title/Abstract]  **AND**  "Therapy, Computer-Assisted"[Mesh] OR "Internet"[Mesh] OR "Mobile Applications"[Mesh] OR "virtual reality"[Mesh] OR "Multimedia"[Mesh] OR web[Title/Abstract] OR technology[Title/Abstract] OR apps[Title/Abstract] OR e-health[Title/Abstract] OR "e-mental health"[Title/Abstract] OR e-therapy[Title/Abstract] OR m-health[Title/Abstract]  **AND**  "Mental health services"[MeSH] OR "Health education"[MeSH] OR Psychotherapy[MeSH] OR Relaxation[MeSH] OR "Self-management"[MeSH] OR Therapy[Title/Abstract] OR Treatment*[Title/Abstract] OR Intervention*[Title/Abstract] OR Program*[Title/Abstract] OR Psychoeducation[Title/Abstract] OR Behaviour*[Title/Abstract] OR Counsel*[Title/Abstract]  **AND**  “randomized controlled trial”[Publication Type] OR randomized[Title/Abstract] OR randomly[Title/Abstract] OR trial[Title/Abstract] |
| **EMBASE** | 'Health Personnel'/exp OR 'Medical staff'/exp OR 'healthcare provider*':ti,ab OR 'healthcare professional*':ti,ab OR 'healthcare worker*':ti,ab OR 'Allied Health':ti,ab OR 'Dentist*':ti,ab OR 'Paramedic*':ti,ab OR 'Nurse*':ti,ab OR 'Therapist*':ti,ab OR 'Physician*':ti,ab OR 'Doctor*':ti,ab  **AND**  'Depression'/exp OR 'mental stress'/exp OR 'Anxiety'/exp OR 'Anxiety disorders'/exp OR 'Depress*':ti,ab OR 'Anxiety':ti,ab OR 'Stress':ti,ab OR 'Burden':ti,ab  **AND**  'Computer-Assisted'/exp OR Internet/exp OR 'Mobile Applications'/exp OR 'virtual reality'/exp OR Multimedia/exp OR 'web':ti,ab OR 'technology':ti,ab OR 'apps':ti,ab OR 'e-health':ti,ab OR 'e-mental health':ti,ab OR 'e-therapy':ti,ab OR 'm-health':ti,ab  **AND**  'Mental health services'/exp OR 'Health education'/exp OR 'Psychotherapy'/exp OR 'Relaxation'/exp OR 'Self-management'/exp OR 'Therapy':ti,ab OR 'Treatment*':ti,ab OR 'Intervention*':ti,ab OR 'Program*':ti,ab OR 'Psychoeducation':ti,ab OR 'Behaviour*':ti,ab OR 'Counsel*':ti,ab  **AND**  'crossover procedure':de OR 'double-blind procedure':de OR 'randomized controlled trial':de OR 'single-blind procedure':de OR (random* OR factorial* OR crossover* OR cross NEXT/1 over* OR placebo* OR doubl* NEAR/1 blind* OR singl* NEAR/1 blind* OR assign* OR allocat*):de,ab,ti OR (rct OR 'randomly allocated' OR 'allocated randomly' OR 'random allocation' OR allocated NEAR/2 random):ab,ti |
| **Cochrane Library** | #1 MeSH descriptor: [Health Personnel] explode all trees  #2 MeSH descriptor: [Medical staff] explode all trees  #3 ("healthcare provider*" OR "healthcare professional*" OR "healthcare worker*" OR "Allied Health" OR "Dentist*" OR "Paramedic*" OR "Nurse*" OR "Therapist*" OR "Physician*" OR "Doctor*"):ti,ab,kw  #4 MeSH descriptor: [Depression] explode all trees  #5 MeSH descriptor: [Anxiety] explode all trees  #6 MeSH descriptor: [Anxiety disorders] explode all trees  #7 MeSH descriptor: [Stress, psychological] explode all trees  #8 ("Depress*" OR Anxiety OR Stress):ti,ab,kw  #9 MeSH descriptor: [Therapy, Computer-Assisted] explode all trees  #10 MeSH descriptor: [Internet] explode all trees  #11 MeSH descriptor: [Mobile Applications] explode all trees  #12 MeSH descriptor: [virtual reality] explode all trees  #13 MeSH descriptor: [Multimedia] explode all trees  #14 (e-health OR “e-mental health” OR e-therapy OR m-health):ti,ab,kw  #15 MeSH descriptor: [Mental health services] explode all trees  #16 MeSH descriptor: [Psychotherapy] explode all trees  #17 MeSH descriptor: [Relaxation] explode all trees  #18 MeSH descriptor: [Self-management] explode all trees  #19 (Therapy OR "Treatment*" OR "Intervention*" OR "Program*" OR Psychoeducation OR "Behaviour*" OR "Counsel*"):ti,ab,kw  #20 #1 OR #2 OR #3  #21 #4 OR #5 OR #6 OR #7 OR #8  #22 #9 OR #10 OR #11 OR #12 OR #13 OR #14  #23 #15 OR #16 OR #17 OR #18 OR #19  #24 #20 AND #21 AND #22 AND #23 in Trials |
| **PsycINFO** | exp Health Personnel/ or ('healthcare provider*' or 'healthcare professional*' or 'healthcare worker*' or 'medical staff' or 'Allied Health' or 'Dentist*' or 'Paramedic*' or 'Nurse*' or 'Therapist*' or 'Physician*' or 'Doctor*').ti,ab.  **AND**  exp Depression/ or exp Anxiety/ or exp Psychological Stress/or ('Depress*' or 'Anxiety' or 'Stress').ti,ab.  **AND**  exp Internet/ or exp Mobile Applications/ or exp virtual reality/ or exp Multimedia/ or (web or computer or technology or apps or e-health or 'e-mental health' or e-therapy or m-health).ti,ab.  **AND**  exp Mental health services/ or exp Health education/ or exp Psychotherapy/ or exp Relaxation/ or exp Self-management/ or (Therapy or 'Treatment*' or 'Intervention*' or 'Program*' or 'Psychoeducation' or 'Behaviour*' or 'Counsel*').ti,ab.  **AND**  (Randomized Controlled Trial or Pragmatic Clinical Trial or Equivalence Trial or Clinical Trial, Phase III).pt. or Randomized Controlled Trial/ or "Randomized Controlled Trial (topic)"/ or Randomization/ or Random Allocation/ or Double-Blind Method/ or Double Blind Procedure/ or Single-Blind Method/ or Single Blind Procedure/ or ((singl* or doubl*) adj (blind* or dumm* or mask*)).ti,ab,hw.or (random or randomly or randomised or randomized).ti,ab. |
| **Web of science** | #1 TI=(Health Personnel OR Medical staff OR healthcare provider* OR healthcare professional* OR healthcare worker* OR Allied Health OR Dentist* OR Paramedic* OR Nurse* OR Therapist* OR Physician* OR Doctor*)  #2 AB=(Health Personnel OR Medical staff OR healthcare provider* OR healthcare professional* OR healthcare worker* OR Allied Health OR Dentist* OR Paramedic* OR Nurse* OR Therapist* OR Physician* OR Doctor*)  #3 #1 OR #2  #4 TI=(Depress* OR Anxiety OR Stress)  #5 AB=(Depress* OR Anxiety OR Stress)  #6 #4 OR #5  #7 TI=(Internet OR Computer OR Mobile Applications OR virtual reality OR Multimedia OR web OR technology OR apps OR e-health OR e-mental health OR e-therapy OR m-health)  #8 AB=(Internet OR Computer OR Mobile Applications OR virtual reality OR Multimedia OR web OR technology OR apps OR e-health OR e-mental health OR e-therapy OR m-health)  #9 #7 OR #8  #10 TI=(Mental health services OR Health education OR Psychotherapy OR Relaxation OR Self-management OR Therapy OR Treatment* OR Intervention* OR Program* OR Psychoeducation OR Behaviour* OR Counsel*)  #11 AB=(Mental health services OR Health education OR Psychotherapy OR Relaxation OR Self-management OR Therapy OR Treatment* OR Intervention* OR Program* OR Psychoeducation OR Behaviour* OR Counsel*)  #12 #10 OR #11  #13 TI=(double-blind OR randomized controlled trial OR random* OR trial)  #14 AB=(double-blind OR randomized controlled trial OR random* OR trial)  #15 #13 OR #14  #16 #3 AND #6 AND #9 AND #12 AND #15 |
| **ProQuest Dissertations & Theses Global** | ti("Health Personnel" OR “Medical staff” OR "healthcare provider*" OR "healthcare professional*" OR "healthcare worker*" OR "Allied Health" OR "Dentist*" OR "Paramedic*" OR "Nurse*" OR "Therapist*" OR "Physician*" OR "Doctor*") OR ab("Health Personnel" OR “Medical staff” OR "healthcare provider*" OR "healthcare professional*" OR "healthcare worker*" OR "Allied Health" OR "Dentist*" OR "Paramedic*" OR "Nurse*" OR "Therapist*" OR "Physician*" OR "Doctor*")  **AND**  ti(Depress* OR Anxiety OR Stress) OR ab(Depress* OR Anxiety OR Stress)  **AND**  ti(“Computer” OR “Internet” OR “Mobile Applications” OR “virtual reality” OR “Multimedia” OR web OR technology OR apps OR e-health OR “e-mental health” OR e-therapy OR m-health) OR ab(“Computer” OR “Internet” OR “Mobile Applications” OR “virtual reality” OR “Multimedia” OR web OR technology OR apps OR e-health OR “e-mental health” OR e-therapy OR m-health)  **AND**  ti("Mental health services" OR "Health education" OR Psychotherapy OR Relaxation OR "Self-management" OR Therapy OR Treatment* OR Intervention* OR Program* OR Psychoeducation OR Behaviour* OR Counsel*) OR ab("Mental health services" OR "Health education" OR Psychotherapy OR Relaxation OR "Self-management" OR Therapy OR Treatment* OR Intervention* OR Program* OR Psychoeducation OR Behaviour* OR Counsel*)  **AND**  ti("double-blind" OR "random* assigned" OR randomly OR randomised OR randomized) OR ab("double-blind" OR "random* assigned" OR randomly OR randomised OR randomized) |
| **Cnki** | (SU % ‘医务人员’ or SU % ‘医疗工作者’ or SU % ‘卫生人员’ or SU % ‘医生’ or SU % ‘医师’ or SU % ‘护士’)  AND  (SU % ‘抑郁’ or SU % ‘焦虑’ or SU % ‘压力’)  AND  (SU % ‘电子’ or SU % ‘电脑’ or SU % ‘互联网’ or SU % ‘手机’ or SU % ‘apps’ or SU % ‘网络’ or SU % ‘多媒体’ or SU % ‘虚拟’ or SU % ‘数字’ or SU % ‘远程’)  AND  (SU % ‘心理’ or SU % ‘心理健康’ or SU % ‘求助’ or SU % ‘心理咨询’ or SU % ‘心理教育’ or SU % ‘放松’ or SU % ‘干预’ or SU % ‘治疗’ or SU % ‘项目’ or SU % ‘工具’ or SU % ‘课程’ or SU % ‘行为’)  AND  (SU % ‘随机对照实验’ or SU % ‘RCT’ or SU % ‘随机’ or SU % ‘实验’) |
| **Wanfang** | 主题:(**"**医务人员**"** or **"**医疗工作者**"** or **"**卫生人员**"** or **"**医生**"** or **"**医师**"** or "护士")  and  主题:("抑郁" or "焦虑" or "压力")  and  主题:("电子" or "电脑" or "互联网" or "手机" or "apps" or "网络" or "多媒体" or "虚拟"or "数字" or "远程")  and  主题:(**"**心理**"** or **"**心理健康**"** or **"**求助**"** or **"**心理咨询**"** or **"**心理教育**"** or **"**放松**"** or **"**干预**"** or **"**治疗**"** or **"**项目**"** or **"**工具**"** or **"**课程**"** or **"**行为**"**)  and  主题:(**"**随机对照实验**"** or **"**RCT**"** or **"**随机**"** or **"**实验**"**) |

## Table 3: Eligibility criteria

| Criteria | Inclusion | Exclusion |
| --- | --- | --- |
| Population | - All studies enrolling healthcare professionals; | - Medical students; - Midwifery students; - Staff with cognitive impairment |
| Intervention | - Examining e-mental health interventions; - E-mental health interventions refer to a series of service that use information technology to maintain and promote people's psychological well-being, including prevention, diagnosis, monitoring, treatment, and management. |  |
| Comparison | - No restrictions will be placed on the type of usual care or active control that is provided. |  |
| Outcomes | - Measuring at least one of the following outcomes: stress, depression and anxiety; - Other outcomes may include burnout, well-being, work engagement, self-efficacy, job satisfaction, and etc. - inappropriate outcomes(no stress, depression, or anxiety) | - Inappropriate outcomes (no stress, depression, or anxiety) |
| Type of design | - Any types of randomized controlled trials | - Non-experimental studies (retrospective or prospective cohort studies;case-control studies;one-group posttest-only design ) - qualitative or reviews |
| Years of publication | - No limit |  |
| Publication type | - Published and unpublished trials | - Abstract only - Book chapter review - Letters - Editorials - Systematic review |
| Language | - No limit |  |

## Table 4: Data extraction table

Author:

Year:

Title:

Doi:

| Setting | |  | | | City/Country | | |  | | | | |
| --- | --- | --- | --- | --- | --- | --- | --- | --- | --- | --- | --- | --- |
| Grant Support | |  | | | | | | | | | | |
| Protocol (Y/N) | |  | | | Registration No. | | |  | | | | |
| Participants | |  | | | Design | | |  | | | | |
| Age | |  | | | | | | | | | | |
| Sample Size | | Total | |  | | | | | | | | |
|  |  | Intervention | |  | | | | | | | | |
|  |  | Control | |  | | | | | | | | |
| Intervention (name) | |  | | | | | | Co-intervention (Y/N) | | | |  |
| Aim of Intervention | |  | | | | | | | | | | |
| Duration of Intervention | |  | | | | | | | | | | |
| Components of Intervention | |  | | | | | | | | | | |
| Comparator | |  | | | | | | | | | | |
| Provider/Self Help | | Intervention | | | | | | Comparator | | | | |
|  |  |  | | | | | |  | | | | |
| Attrition Rate | |  | | | ITT (Y/N) | | |  | | | | |
|  |  |  |  |  | Missing Data (Y/N) | | |  | | | | |
| **Outcomes:** | | | | | | | | | | | | |
| Dichotomous | Measures: | | Time-point | Intervention | | | | | Control | | | |
|  |  |  |  | Event | | Total | | | Event | | Total | |
|  | 1. Outcome No. | |  |  | |  | | |  | |  | |
|  | 2. Outcome No. | |  |  | |  | | |  | |  | |
|  | 3. Outcome No. | |  |  | |  | | |  | |  | |
| Continuous | Measures: | | Time-point | Intervention | | | | | Control | | | |
|  |  |  |  | Mean | SD | | Total | | Mean | SD | | Total |
|  | 1. Outcome No. | |  |  |  | |  | |  |  | |  |
|  | 2. Outcome No. | |  |  |  | |  | |  |  | |  |
|  | 3. Outcome No. | |  |  |  | |  | |  |  | |  |

## Table 5: Characteristics of included studies

| Author/  year/country | Setting | Design  RCT | Population | Age, m±SD/n,% | Sample size | Intervention | Control | Attrition rate (%) | ITT/MDM | Protocol/Registration/Grant support/Ethic review |
| --- | --- | --- | --- | --- | --- | --- | --- | --- | --- | --- |
| Hui Grace Xu et al.  (2022)Australia | Emergency department in hospital | 2-arm | Medical staff | ＜30:61;30-39:37;40-49:30;≥50:20 | T:148;  I:74;  C:74 | App-guided mindfulness practice (Headspace app) | Waitlist control | 35.14 | Y/Y | Y/Y/Y/Y |
| Qingqing Wang  (2021)China | ICUs in tertiary hospital | 2-arm | Nurses | 20-30:52;31-40:47;＞40:5 | T:104;  I:52;  C:52 | Online mindfulness-based stress reduction training | No treatment | 0.00 | NR/Y | NR/NR/NR/Y |
| Shuying Chang et al.  (2021)China | Isolation ward in hospital | 2-arm | Nurses | ≤30:17;31-40:38;≥41:7 | T:62;  I:31;  C:31 | Network Structured Group Psychological Intervention | Active control | 0.00 | NR/NR | NR/NR/Y/Y |
| Ewelina Smoktunowicz et al.  (2021)Poland | Public health care institutions | 4-arm | Medical professionals | 36.21±10.18 | T: 1240;  I: 622;  C:618 | Resource-based internet intervention (Med-Stress website) | Active control | 82.15 | Y/Y | Y/Y/Y/Y |
| Jochen Profit et al.  (2021)USA | NICU in hospital | 2-arm | Healthcare workers | NR | T:481;  I:182;  C:299 | Web-based implementation(WISER) | Waitlist control | 53.43 | NR/Y | NR/NR/Y/Y |
| Kotaro Imamura et al.  (2021)Vietnam | Hospital | 3-arm | Nurses | IA:33.7±7.3;IB:32.8±6.6;C:32.8±6.4 | T:951;  IA:317;  IB:316;  C:316 | Smartphone-based stress management programs (program A: a free-choice, multimodule stress management;program B:a fixed order,internet cognitive behavioral therapy) | TAU | 8.20 | N/Y | Y/Y/Y/Y |
| Maria Antònia Fiol-Deroque et al.  (2021)Spain | Hospital | 2-arm | Healthcare workers | 41.37±10.4 | T:482;  I:248;  C:234 | Mobile phone-based intervention (PsyCovidApp) | Active control | 9.54 | Y/NR | Y/Y/Y/Y |
| Berna Dincer et al.  (2021)Turkey | COVID-19 department in hospital | 2-arm | Nurses | 33.45±9.63 | T:72;  I:35;  C:37 | Online form of emotional freedom techniques(EFT) | No treatment | 10.00 | NR/NR | NR/Y/N/Y |
| Guojun Xie  (2020)China | Hospital | 3-arm | Nurses | 30.08±9.58 | T:180;  I:60;  CA:60;  CB:60 | Intemet-delivered Cognitive Behavioral Therapy (MoodGYM) | No treatment | 5.00 | NR/NR | NR/NR/NR/Y |
| Chuanfang Wu et al.  (2020)China | Hospital | 2-arm | Pregnancy nurses | I:29.84±4.66;C:31.92±4.28 | T:76;  I:38;  C:38 | Mobile phone APP guided mindfulness training based stress reduction(MBSR) | TAU | 0.00 | NR/NR | NR/NR/Y/Y |
| Chunmei Wang et al.  (2020)China | Hospital | 2-arm | Nurses | 28.99±8.63 | T:100;  I:50;  C:50 | Internet-based mindfulness based stress reduction therapy(MBSR) | TAU | 0.00 | NR/NR | NR/NR/NR/Y |
| Kaiqin Deng et al.  (2020)China | Hospital | 2-arm | Nurses | I:20-30:16(37);31-40:12(28);41-50:15(35);C:20-30:15(36);31-40:13(31);41-50:14(33) | T:85;  I:43;  C:42 | Internet-based unstructured group counseling(UGC) | TAU | 0.00 | NR/NR | NR/NR/NR/Y |
| Keith G Lambert et al.  (2020)USA | One urban academic teaching hospital and one community hospital | 2-arm | Emergency medicine personnel | 37±10 | T:36;  I:21;  C:15 | Phone-based meditation application | No treatment | 58.33 | Y/Y | Y/Y/Y/Y |
| Pádraic J. Dunne et al.  (2019)Ireland | Hospital | 2-arm | Emergency medicine physicians | NR | T:58;  I:29;  C:29 | Attention-based training (ABT) program | Waitlist control | 27.59 | N/Y | Y/Y/NR/Y |
| Erin G. Mistretta et al.  (2018)USA | A large research hospital and medical center | 3-arm | Healthcare workers | 46.0±12.6 | T:60;  IA:29;  IB:19;  C:15 | Smartphone-delivered resiliency-based intervention | Waitlist control | 0.00 | Y/NR | NR/NR/Y/Y |
| Peter M. Gollwitzer et al.  (2018)Germany | Hospital; residential care;nursing home;psychiatric institutions;day hospital;rehabilitation centers;not specified institutions | 3-arm | Nurses | 40.22±10.18 | T:129;  I:41;  CA=47;  CB:41 | Internet-bas ed  Intervention Called  Mental  Contrasting  with  Implementa tion intentions (MCII) | No treatment | 18.60 | Y/Y | NR/NR/Y/Y |
| Rebekah K. Hersch et al.  (2017)USA | Hospital | 2-arm | Nurses | 41(22,65) | T:104;  I:52;  C:52 | Web-based stress management program(BREATHE) | Waitlist control | 13.46 | Y/Y | Y/NR/Y/Y |
| Yuchen Huang  (2015)China | Tertiary hospital | 2-arm | Nurses | NR | T:170;  I:88;  C:82 | Solution-focused approach intervention in the mobile internet | No treatment | 0.00 | NR/Y | NR/NR/NR/Y |
| Vivian Low et al.  (2015)USA | Community hospital | 2-arm | Female health care workers | 52±6.3 | T:57;  I:28;  C:29 | Worksite program | TAU | 71.93 | Y/NR | Y/NR/Y/Y |
| Linda Bolier et al.  (2014)Netherlands | One large academic medical centre | 2-arm | Nurses and allied health professionals | 40±11.9 | T:1140;  I:579;  C:561 | Workplace mental health promotion online | Waitlist control | 81.75 | Y/Y | Y/Y/Y/Y |

Note.

SD: standard deviation

ITT: intent to treat

MDM: missing data management

RCT: randomized controlled trial

T: total

I: intervention

C: control

TAU: treatment as usual

NR: not reported

## Table 6: Description of e-mental health intervention

| Author/  year/country | Intervention (name) | Content of intervention | Intervention duration | Follow-up | Outcome measure |
| --- | --- | --- | --- | --- | --- |
| Hui Grace Xu et al.  (2022)Australia | App-guided mindfulness practice (Headspace app) | App-guided mindfulness | 4 weeks, 10 min daily | 3 months | Stress:Perceived Stress Scale (PSS-10) |
| Qingqing Wang  (2021)China | Online mindfulness-based stress reduction training | Implement mindfulness decompression network course through Dingding software, including mindfulness theory explanation and mindfulness practice | 8 weeks, 2h/week | NR | Stress:Source of nurses’ work pressure-32 |
| Shuying Chang et al.  (2021)China | Network Structured Group Psychological Intervention | Carry out network structured supportive group psychological counseling activities through Tencent video software, including emotional expression; rebuild self-confidence and cherish the present; looking ahead | Once/2d, 1.5h/time | 13d | Anxiety:Generalized Anxiexy Disorde-7 (GAD-7)  Depression:Patient Health Questionaire (PHQ -9) |
| Ewelina Smoktunowicz et al.  (2021)Poland | Resource-based internet intervention (Med-Stress website) | Med-Stress is a self-guided internet intervention. Obligatory modules include Self-efficiency enhancement; Perceived social support enhancement. Optional modules include Relaxation; mindfulness; cognitive restructuring; lifestyle. | 6 weeks/once a week/To complete all tasks within each exercise, participants needed up to 1.5 hours | 6 months | Stress:Perceived Stress Scale (PSS-14)  Depression:Patient Health Questionaire (PHQ -9) |
| Jochen Profit et al.  (2021)USA | Web-based implementation(WISER) | Participants were invited to view modules by mobile or email. WISER is comprised of six guided well-being modules combining educational material with practice-based learning: gratitude; three good things; awe; random acts of kindness; identifying and using signature strengths and relationship resilience. | 10-17 min daily; 10 days per month | 6 months | Depression:Center for epidemiological studies depression scale(CES-D10) |
| Kotaro Imamura et al.  (2021)Vietnam | Smartphone-based stress management programs (program A: a free-choice, multimodule stress management;program B:a fixed order,internet cognitive behavioral therapy) | Two smartphone-based six-module stress management programs were used. Program A included behavioral activation (module 1), cognitive restructuring (module 2), problem-solving (module 3), assertiveness (module 4), self-compassion (module 5), and job crafting (module 6). Participants chose one module per week in any order they preferred. Program B included six modules that provide CBT-based stress management skills, including a transactional model of stress and coping (module 1), self-case formulation based on cognitive behavioral model (module 2), behavioral activation (module 3), cognitive restructuring (module 4), cognitive restructuring and relaxation (module 5), and problem-solving (module 6). The six modules were presented in a fixed order, with one module accessible per week, from module 1 to module 6. | 6 modules/one module per week/about 15 minutes to complete each module/complete all modules within 10 weeks | 3 months, 7 months | Anxiety:Vietnamese version of Depression Anxiety and Stress Scale(DASS21-A)  Depression:Vietnamese version of Depression Anxiety and Stress Scale(DASS21-D) |
| Maria Antònia Fiol-Deroque et al.  (2021)Spain | Mobile phone-based intervention (PsyCovidApp) | An app intervention called PsyCovidApp, based on cognitive-behavioral therapy and mindfulness approaches, included written and audiovisual content targeting four areas: emotional skills, healthy lifestyle behavior, work stress and burnout, and social support. | 2 weeks | NR | Stress:Depression Anxiety Stress Scale (DASS-21)  Anxiety:Depression Anxiety Stress Scale (DASS-21)  Depression:Depression Anxiety Stress Scale (DASS-21) |
| Berna Dincer et al.  (2021)Turkey | Online form of emotional freedom techniques(EFT) | A brief online form of emotional freedom techniques(EFT). It conducted using ZOOM. Steps: (1) Identify an anxiety-evoking issue and determine the SUD level. (2) Creating a personal acceptance and reminder statement in the general form of "I accept myself despite this…" (3) Tapping seven times on each acupressure point. (4) After tapping these points, the affirmation/reminder statement is repeated. (5) A sequence of physical movements and vocalizations called “The Nine Gamut Procedure” is carried out. (6) Steps 3 and 4 are repeated. (7) Another SUD rating is given. | approximately 20 min/a single session | NR | Stress:Subjective units of distress scale(SUD)  Anxiety:State-trait anxiety scale-20 |
| Guojun Xie  (2020)China | Intemet-delivered Cognitive Behavioral Therapy (MoodGYM) | The training program consists of five modules, including feeling module, thinking module, changing distorted ideas module, reducing stress module and relationship module. Each module includes assessment, information, emotional exercise, case demonstration and test. | 30-45 minutes each time/once a week/2 months | NR | Anxiety:Depression Anxiety Stress Scale (DASS-21)  Depression:Depression Anxiety Stress Scale (DASS-21) |
| Chuanfang Wu et al.  (2020)China | Mobile phone APP guided mindfulness training based stress reduction(MBSR) | Mindfulness training includes meditation pre-school classes; basic meditation practice; relaxation meditation practice; workplace meditation practice; meditation practice during pregnancy; thanksgiving meditation practice; life meditation practice. | 30-60min daily/8 weeks | NR | Anxiety:Self-reporting inventory (SCL-90)  Depression:Self-reporting inventory (SCL-90) |
| Chunmei Wang et al.  (2020)China | Internet-based mindfulness based stress reduction therapy(MBSR) | Mindfulness training includes mindfulness breathing, mindfulness meditation, body scanning, mindfulness stretching, mindfulness diet, mindfulness yoga, etc. | 30-40 minutes/time, 3 times/week, 4 weeks | NR | Anxiety:Self-Rating Anxiety Scale(SAS-20)  Depression:Self-Rating Depression Scale(SDS-20) |
| Kaiqin Deng et al.  (2020)China | Internet-based unstructured group counseling(UGC) | It uses Tencent conference as its main software; Including psychological topics, management topics, patient topics, technical topics, life topics, etc. | 125 minutes/time, 1 time/2 days, 10 days | NR | Stress:Chinese perceived stress scale (CPSS) |
| Keith G Lambert et al.  (2020)USA | Phone-based meditation application | Intervention subjects were provided instructions on downloading the application with twelve guided meditations. | 3.5 to 21 min, use the application weekly, 90 days | 6 months | Stress:Perceived Stress Scale (PSS)  Anxiety:Beck Anxiety Inventory (BAI)  Depression:Beck Depression Inventory (BDI) |
| Pádraic J. Dunne et al.  (2019)Ireland | Attention-based training (ABT) program | Under help of a bespoke smart phone app, ABT practice involved repeatedly focusing one’s attention on a chosen non-English phrase (maranatha). | 20 min, twice daily, the combined target was two 20-minute sessions over 7 days (280 min in total) | 2 months | Stress:Depression Anxiety Stress Scale (DASS-21)  Anxiety:Depression Anxiety Stress Scale (DASS-21) |
| Erin G. Mistretta et al.  (2018)USA | Smartphone-delivered resiliency-based intervention | Topics of smartphone resilience training includes sleep (fall sleep faster and feel more refreshed); happiness and positivity (be happier); energy and focus (boost my energy and focus); productivity (get things done); mixture of topics (feel less stressed). | 6-week trail/every 7 to 10 days, participants were prompted to select one of four possible topics that they wanted to focus on for the next week | 3 months | Stress:Depression Anxiety Stress Scale (DASS-21)  Anxiety:Depression Anxiety Stress Scale (DASS-21)  Depression:Depression Anxiety Stress Scale (DASS-21) |
| Peter M. Gollwitzer et al.  (2018)Germany | Internet-bas ed  Intervention Called  Mental  Contrasting  with  Implementa tion intentions (MCII) | The MCII strategy was summarized in the four questions: What is the best possible outcome today of my wish to have less stress? What is the main obstacle today to fulfilling this wish? How can I act to overcome this obstacle? What is my if-then plan today? | Once a day, 3 weeks | NR | Stress:Overall stress index Perceived stress questionnaire(PSQ-20) Physical symptoms subscale of the Burnout Screening Scales II inventory (BOSS II-10) |
| Rebekah K. Hersch et al.  (2017)USA | Web-based stress management program(BREATHE) | The program consists of seven modules for nurses and an additional module for nurse managers. The seven modules are: welcome and introduction; assess your stress; identify stressors; manage stress; avoid negative coping; your mental health; the manager’s role. | Participants were encouraged to use the program as often as they want over a three-month period | NR | Stress:Nursing stress scale(NSS) |
| Yuchen Huang  (2015)China | Solution-focused approach intervention in the mobile internet | Testers used various techniques during the interventions including actively-guided opening questions, complicated formulations, positive intervention tactics and Solution-Focused skills. Test subjects was presented with questions and replied through cell-phone app. | Everyday, 21 days (3 weeks) | NR | Stress:Source of nurses’ work pressure-35 |
| Vivian Low et al.  (2015)USA | Worksite program | All participants were offered classes (weight/diet, stress, exercise, and smoking cessation) and gym access. The intervention group participants were told that they would receive weekly communication via phone or e-mail, incorporating goal-setting and suggestions for overcoming obstacles. | 6 months | 1 year | Stress:Cohen stress scale |
| Linda Bolier et al.  (2014)Netherlands | Workplace mental health promotion online | In the case of negative screening on mental health complaints, participants were invited to take an online course, including psyfit, colour your life, strong at work, don’t panic online, drinking less. | The study included 5 online interventions, every intervention had different duration. Psyfit: one or more 4 weeks. Colour your Life: 8 weeks/ 30 mins weekly + a booster session. Strong at work: 8 weeks/ 30 mins weekly +a booster session. Don't panic Online: 8 weeks/ 30-min sessions, 6 times. Drinking Less: 6 weeks/ example for 10 min a day. | 6 months | Anxiety:Brief Symptom Inventory (BSI)  Depression:Brief Symptom Inventory (BSI) |

Note.

NR: not reported

## Figure 1 Risk of bias within studies summary

##
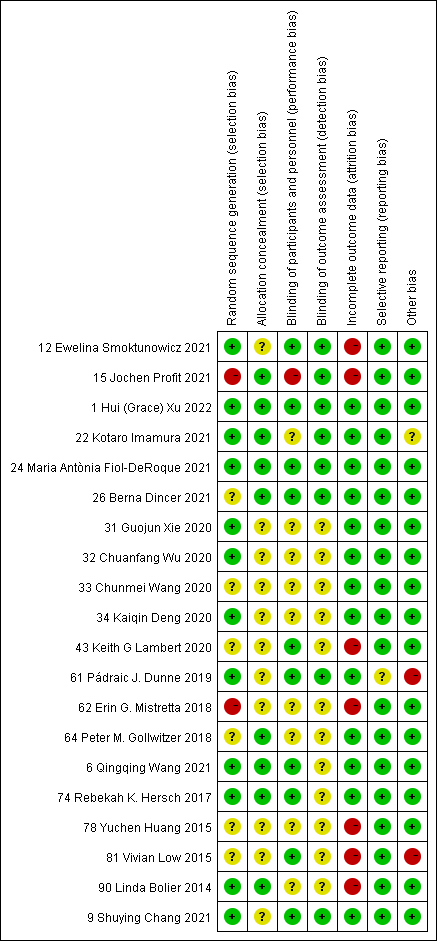


## Table 7: Summary of Findings table

| **Certainty assessment** | | | | | | | **№ of patients** | | **Effect** | | **Certainty** | **Importance** |
| --- | --- | --- | --- | --- | --- | --- | --- | --- | --- | --- | --- | --- |
| **№ of studies** | **Study design** | **Risk of bias** | **Inconsistency** | **Indirectness** | **Imprecision** | **Other considerations** | **stress** | **placebo** | **Relative (95% CI)** | **Absolute (95% CI)** |  |  |
| **stress** | | | | | | | | | | | | |
| 11 | randomised trials | not serious | very serious^a^ | not serious | not serious | none | 627 | 636 | - | SMD **1.21 lower** (1.85 lower to 0.56 lower) | ⨁⨁◯◯ Low | IMPORTANT |
| **anxiety** | | | | | | | | | | | | |
| 10 | randomised trials | not serious | very serious^b^ | not serious | not serious | none | 844 | 895 | - | SMD **0.83 lower** (1.29 lower to 0.37 lower) | ⨁⨁◯◯ Low | IMPORTANT |
| **depression** | | | | | | | | | | | | |
| 9 | randomised trials | serious^c^ | serious^d^ | not serious | not serious | none | 809 | 858 | - | SMD **0.3 lower** (0.49 lower to 0.11 lower) | ⨁⨁◯◯ Low | IMPORTANT |

**CI:** confidence interval; **SMD:** standardised mean difference

Explanations

a. I square value (96%) is very large.

b. I square value (94%) is very large.

c. Majority of allocation concealment (67%), blinding of participants and personnel (67%), blinding of outcome assessment (67%) were unclear.

d. I square value (64%) is large.


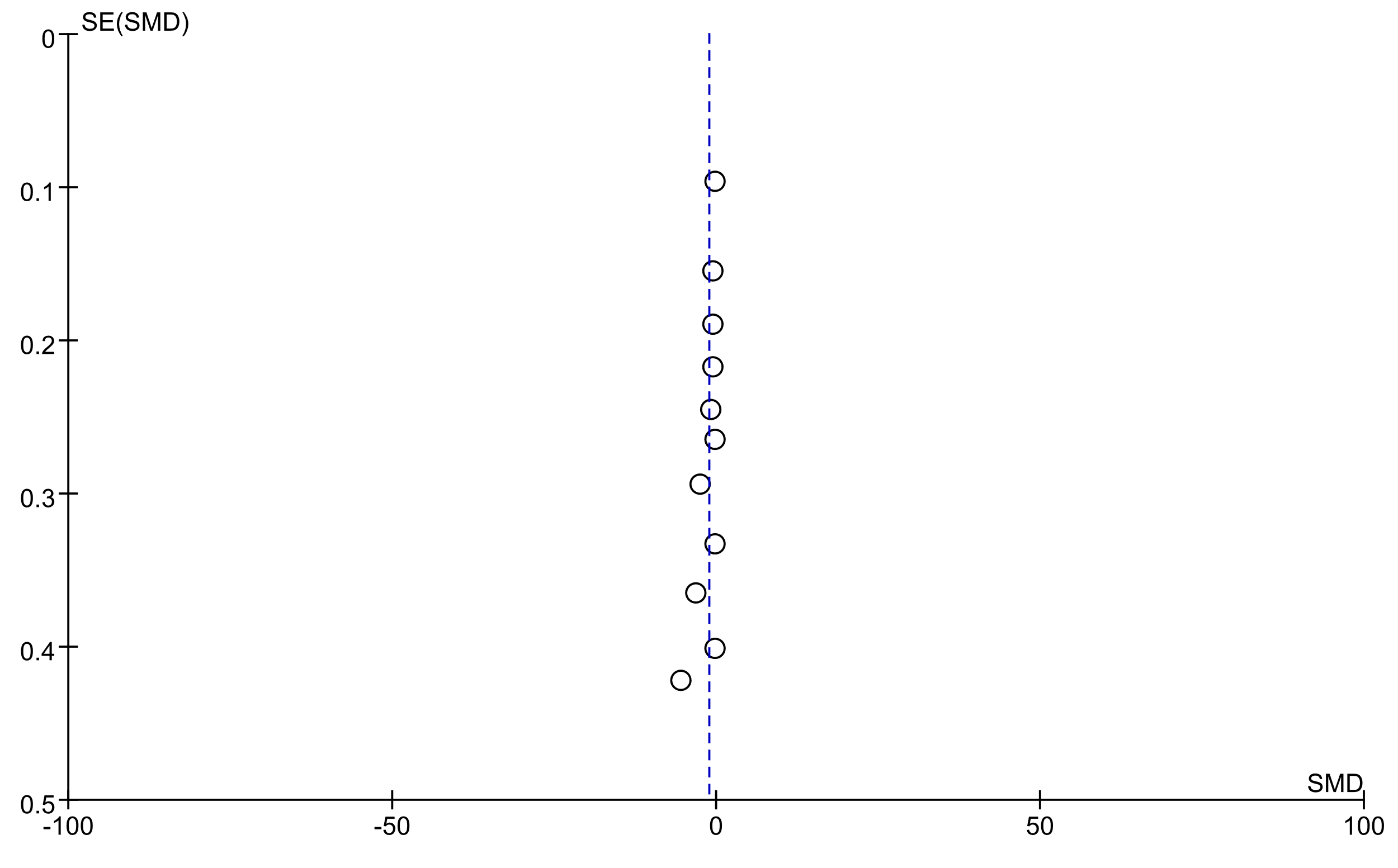


## Figure 2 Funnel plot of stress


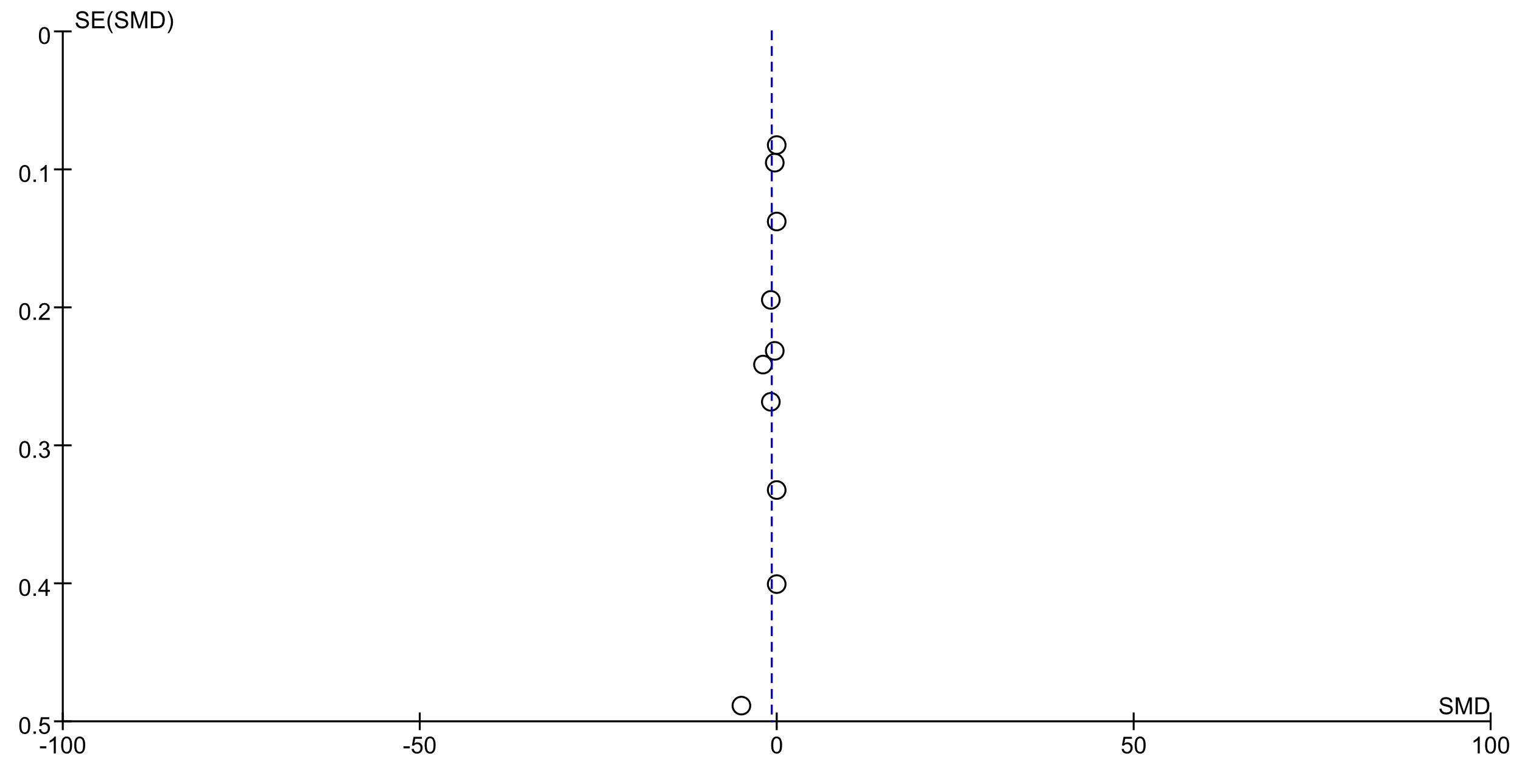


## Figure 3 Funnel plot of anxiety
